# The Affective Semiosis of the Hypoglycemic Symptom in Type 1 Diabetes Mellitus

**DOI:** 10.1007/s12124-024-09889-x

**Published:** 2025-02-17

**Authors:** Juan José Cleves-Valencia, Mónica Roncancio-Moreno, Angela Uchoa Branco

**Affiliations:** 1 Universidad del Valle, Santiago de Cali, Colombia; 2 Universidade de Brasília, Brasília, Brazil

**Keywords:** Type 1 diabetes mellitus, Hypoglycemia, Cultural psychology, Case studies, Semiotic mediation, Affect

## Abstract

Type 1 Diabetes Mellitus (T1DM) involves a complex treatment because its daily management requires patients to maintain a delicate balance to avoid the symptomatic discomfort of hypoglycemia. Although hypoglycemia has been studied from a biomedical perspective, there is limited research related to the meaning construction and affective regulation processes associated with it. The present study aims to understand the affective-semiotic processes involved in the hypoglycemic symptom of T1DM by analyzing its dynamic organization and affective-semiotic regulation. Methodologically, an idiographic perspective was chosen through the microgenetic analysis of in-depth interviews in a case study. The main results indicate that the initial sensation of physical discomfort is merely the starting point of a wider semiotic network that hyper-generalizes and expands itself towards broader aspects of life and identity. Life projections, whether short- or long-term, are mediated by the fear of the symptom, which, in turn, can shape one´s perception of life priorities in life and involves the potential to shape one’s future in terms of the ability to maintain health. It is proposed that the hypoglycemic symptom, as a set of experiences with the capacity to destabilize the individual, should be considered in order to: (a) Evaluate how each one is affected through affective-semiotic fields of meaning directly linked to the perception of oneself as a subject, (b) Reduce the uncertainty associated with the body’s vulnerability and contingency and, (c) Foster a greater personal empowerment with respect to the disease.

## Introduction

Type 1 Diabetes Mellitus (hereinafter T1DM) requires a complex and highly demanding medical treatment for those who live it in the intimate and public spheres of social life. It is a chronic disease whose diagnosis implies processes of meaning-making that allow its sufferers to maintain a feeling of relative continuity in themselves and their relationships with the surrounding universe of people and objects. Rooted in the body, this condition presents challenges regarding desire and the sense of control that are strong enough to disrupt the living situation of the individual who, after the diagnosis, has partially lost “the compass” and “the map” with which they had oriented their life (Frank, 1995). In this sense, understanding the relationships established with the disease inevitably implies addressing the processes of dynamic elaboration of meanings to understand how subjects continuously reinterpret and negotiate the meanings introduced by the disease as they constantly deal with the specificities of their treatment.

In most cases, T1DM occurs when the defense system has attacked the Beta Cells in the pancreas, so the patient’s body can no longer perform the function of insulin secretion. In the long term, due to the dysregulation of glucose levels and its permanent circulation in the blood, this condition can lead to the deterioration of tissues whose main affections are manifested in organs such as the kidneys, eyes, heart, and feet. To maintain glucose at an adequate level, people need continuous exogenous supplies of insulin through rapid- and long-acting injection schedules, or through automatic mechanisms such as insulin pumps (American Diabetes Association [ADA], 2023; International Diabetes Federation [IDF], 2021).

However, the complexity and high demands of treatment also consist of the fragile balance that the patient must maintain to avoid both hyperglycemia and hypoglycemia (Cleves-Valencia & Ocampo-Cepeda 2018; Coffen, 2009). In the first case, the symptoms usually experienced are blurred vision, thirst, physical exhaustion, hunger and constant urination. In the second, they are sweating, tremors, a feeling of weakness and dizziness. The specialized literature reports that, in the daily management of diabetes, patients often choose to avoid hypoglycemia. Studies explain that an alarm is triggered at the physiological level, giving the body a sense of urgency and hypervigilance, and that prevention or rapid correction of hypoglycemia involves physiological and behavioral defenses (International Hypoglycaemia Study Group, 2015). On the other hand, associated with this symptomatology is a degree of embarrassment because of how disruptive it can be in social situations (Datye et al., 2019).

The literature on this topic indicates that hypoglycemia has been studied with the aim of achieving glycemic objectives, optimizing quality of life, or contributing to the management of diabetes, from interdisciplinary orientations that use the conceptual tools of the healthcare field. Thus, for example, a recent study identified that adult patients experience emotional, social, cognitive, and behavioral impacts of hypoglycemia on their relationships, occupations (work, studies), leisure, daily life, sleep, sex life, and health (physical, mental). These impacts may be short-term or associated with the ongoing risk of hypoglycemia. In this sense, research shows that hypoglycemic episodes cause disruptions, limit participation in activities, and cause exhaustion and concentration difficulties. Furthermore, living with the risk of hypoglycemia involves fear and worry, leading patients to sometimes opt for higher glucose levels, and to feel limited in their spontaneity (Chatwin et al., 2021). Another more recent study identified the extent of fear regarding hypoglycemia, and how it negatively affects quality of life and diabetes management. Its authors indicate the existence of an instrument to assess the fear of hypoglycemia, validated in patients with T1DM and frequently used in research settings. The study reports that 30% of participants had a high fear of hypoglycemia and that those with higher levels of fear also showed higher scores on glycosylated hemoglobin, frequency of hypoglycemic events, and impaired awareness of hypoglycemia (Peter et al., 2023).

Although there are valuable theoretical developments from various orientations of cultural psychology that are applicable to the analysis presented here (De Picione et al., 2019; Hermans, 2022; Kleinman et al., 1978; Salvatore & Zittoun, 2011; Valsiner, 2021; Zittoun, 2006), to our knowledge, the specialized literature has not yielded works specifically addressing meaning-making processes in chronic, incurable diseases, with multicomponent and self-administered treatments such as T1DM. This gap becomes even more noticeable in the specific context of research tackling the potent affects involved in the experience of the hypoglycemic symptom. Therefore, we consider it of utmost importance to make an empirical contribution to the field of affective regulation of the semiosis of this symptom, which would also help expand the tendency observed by some pioneers of cultural psychology, who have noted that this emerging area has traditionally been oriented to the study of issues related to behavior and cognition (Branco & Valsiner, 2010). Thus, we are interested in introducing the notion of affect, understood as semiotically mediated hierarchical fields, to explore what the experience of the hypoglycemic symptom entails in T1DM.

The dynamic nature of semiosis and human development processes are based on tensions, ruptures, continuous negotiations of meanings, and the semiotic regulation of dynamic affective fields. This perspective allows an understanding of how human beings, individually and collectively, “construct their worlds,” making it possible to grasp the specificity and variability of the meanings elaborated in individual and relational contexts. Furthermore, the application of its analytical tools allows researchers to overcome the limitations in psychological practice derived from the predominant emphasis on the cognitive aspects of human behavior, characteristic of some widely accepted approaches in the study of health (Cleves-Valencia et al., 2024; Crossley, 2000; Marks, 2002; Ogden, 2023; Roncancio-Moreno et al., 2023).

## The Dynamic Nature of Semiosis and the Processes of Human Development

The semiotic-cultural psychology perspective holds that human beings continuously create meanings in their relationship with the world. One of its central concepts is mediation, which establishes that between objective and subjective reality, there is a transit facilitated by a semiotic order. Signs enable the meaning maker to distance themselves from the immediate context of the “here and now”, which quickly moves from the present to the past. By understanding human experience as a continuous flow of consciousness, semiotically mediated, it is possible to grasp the processes involved in constructing novelty in the human mind. Furthermore, the mediation of signs is not limited to representing the world but involves a constant and transformative movement between different sign structures, whose dynamics allow the emergence and transformation of affective tensions in a flexible and adaptive way (Branco & Valsiner, 2010; Valsiner, 2014).

The construction of meanings occurs in irreversible time and depends on the active role of the individual and the simultaneous emergence of the context. The universal characteristic of meaning construction is its dependency on context, that is, on specific local conditions. Any meaning is based on a broader and more general context that gives it significance. But the subject creates the dynamics of the specific context, which emerges together with the signs that signify the current situation. This can occur quickly, as in the case of signs used to give meaning and structure to the context in real time. In turn, each context is unique and specific, resulting in a great variability of ways in which meaning is expressed (Branco & Valsiner, 2010; Valsiner, 2014). It is also worth emphasizing that meaning processes - a central dimension for the semiotic cultural psychology approach - are deeply guided by affectivity and are continually negotiated by experiences of communication and interaction with things in the world. Memories of the past, experiences of the present, and expectations for the future play an important role in these continuous processes of meaning making and re-signification that occur at both the level of personal culture (subject) and collective culture (society).

## Affective-semiotic Fields and the Dynamic Regulation of the Self

The central proposition of the semiotic dynamics perspective is that in interacting with culture, the individual creates signs that regulate the very process of acting in irreversible time. The function of signs is always future-oriented; both in their immediate impact, which converts the immediate future into a new present; and in their general orientation towards the encounter with similar situations. Now, although the use of signs is future-oriented, human beings access different traces of the signs of the past (Branco & Valsiner, 2010; Valsiner, 2021). The model of Self-regulation through affective-semiotic fields, in turn, is widely recognized in Cultural Psychology. It fundamentally posits various levels of differentiation of affect, ranging from bodily sensations, to words, to hyper-generalized levels of human experience. From this approach, affect is understood as a term encompassing emotions and feelings. Emotions are typically represented as categories that correspond to punctual signs. Feelings, in turn, are understood as affective phenomena that function as broader fields escaping precise definitions in terms of language. While emotions are distinguished by being discrete, punctual categories, accessible to carry out processes of abstraction, discursive operations, and generalization; feelings are characterized by their diffuse nature. Nonetheless, it is vital to understand that feelings cannot be strictly categorized, as their nature -like that of an affective flow- eludes the constraints of categorization. (Branco & Valsiner, 2010; Lehmann, 2024; Valsiner, 2007, 2014, 2021).

In the perspective of affective-semiotic regulation, the phenomena of affect are presented as punctual signs (for instance, the emotion categorized as anger) and as signs that function as affective-semiotic fields characterized by their dynamic nature, forming hierarchies that, depending on context and time, may change. The model explains how meanings acquire different levels of generalization and appropriation in the flow of experience. Through exchanges with culture, the subject creates hyper-generalized affective-semiotic fields, produced by powerful affects capable of regulating other levels (Roncancio-Moreno et al., 2019). A crucial aspect is the recognition of the limited access of human consciousness to parts of the system, both at the level of immediate sensation and at the level of hyper-generalized affective fields, as the affective life of human beings at these levels is unverbalizable. Hyper-generalization renders these fields very powerful over the psyche -as is the case with values and prejudices- but this dimension of power cannot be represented verbally. This level of hierarchical integration is characterized as “nebulous” and “diffuse”. Its meanings have transcended their original context of emergence, commanding the subject’s intrapersonal world and their concrete actions (Branco & Valsiner, 2010).

At both intentional and unintentional levels, signs regulate the appearance, proliferation, and disappearance of other signs in at least three ways. First, the hyper-generalization of affect leads to the construction of semiotic terminators, which can inhibit the emergence or action of certain signs. Thus, a superordinate field operates as a semiotic terminator of the subordinate sign, giving rise to phenomena of negation, displacements, repression, etc. Second, there are semiotic enhancers, which are signs that reorganize the quality of experience in a catastrophic way. These are generalized and hyper-generalized signs that substantially alter the tone of the feeling. Third, the subject can intentionally potentiate affective fields to promote signs that generate certain feelings, which may be “positive” or “negative” (Branco & Valsiner, 2010; Valsiner, 2014).

Recent revisions of this model add that affective processes unfold in both chronological and phenomenological time. In other words, although these processes must be understood historically, the revisions add that the affective world often follows a phenomenological order. That is, the way in which human beings construct meanings about their experiences is not always sequential but is dictated by the felt intensity of moments in our lives (Dilthey, 1978). Some situations have a greater impact than others, and memories or anticipations of the future are constantly mixed with perceptions of the here and now (Lehmann, 2019; Lehmann & Murakami, 2024).

This paper proposes that the model of affective-semiotic regulation of the Self, through the dynamic generation of affective-semiotic fields and its recent revisions, is promising for elucidating the intriguing questions posed to psychological practice regarding the creation of zones for the joint co-construction of meanings that enables individuals to understand the internal and external realities involved in the experience of the hypoglycemic symptom in a chronic disease such as T1DM (Salvatore & Gennaro, 2015; Salvatore & Zittoun, 2011; Roncancio-Moreno et al., 2023). The hypoglycemic symptom dynamically integrates bodily sensations, diffuse affects, discrete emotions, powerful meanings about existence, as well as the dynamic interplay between actual and virtual temporal dimensions (Branco & Valsiner, 2010; Lehmann & Murakami, 2024; Valsiner, 2014, 2021). Thus, our research question is: What does experiencing hypoglycemic symptoms in everyday life imply affectively for patients with T1DM as they attempt to manage their health condition? To answer this question, we aim to understand the affective-semiotic processes involved in the hypoglycemic symptom of T1DM by analyzing its dynamic organization and affective-semiotic regulation, to propose its subjective implications for future coping, and highlight some implications for medical and psychological interventions in patient treatment.

## Methodology

The study follows an idiographic, subjectivist approach, which views reality as a singular and culturally constructed phenomenon, semiotically mediated, and in relation to others (Branco, 2007; Salvatore & Valsiner, 2010). The results are reported through the presentation of a single case. This methodological choice aligns with the epistemological core of cultural psychology, where individual cases serve as rich contexts for uncovering broader psychological and cultural dynamics (De Picione, 2015; Valsiner, 2023). The analysis of singular cases allows researchers to identify “general processes within the particular”, reflecting the intricate interplay between individual subjectivity and shared cultural meanings (Valsiner, 2014). Furthermore, the idiographic approach can address any psychological object by situating it within its contextual field and temporal development. Psychological objects are conceptualized as “non-existing objects”, meaning abstractions derived from the researcher’s modeling process rather than from direct phenomenological occurrences (Salvatore & Valsiner, 2010). By deeply engaging with Lucía’s case, we demonstrate how the interplay between individual and cultural meanings in specific contexts illuminates broader theoretical frameworks, affirming the idiographic approach’s role in uncovering the dynamic processes that define human psychological experiences.

### Participants

The selection of the participant -Lucia- was based on her expressed and inferred willingness to participate, the potential richness of the qualitative data she could provide, and the quality of the encounter and relationship established with the researcher during the first interview.

### Co-construction of Information

The information was co-constructed with the participant through in-depth interviews. Scripts were designed with flexible conversation topics. Each script contained initial questions that encouraged open verbal expression, guided by non-directive principles. These scripts were validated by an expert psychotherapist with more than 35 years of experience. After each interview, the scripts were modified to include clarifying, more specific questions and to address emerging themes. Personal field notes were taken to document the researcher’s questions, interpretations, intuitions, and reflections on the participant’s responses. The interviews were recorded using the Easy Voice Recorder application for smartphones. The interviews were fully transcribed using Otranscribe software. The recording unit was the participant’s verbalizations. Analytically, it was identified when the interviews did not provide more information (saturation) and when it was sufficient regarding the research objective.

### Procedures and Information Analysis

A microgenetic analysis was conducted. The theoretical framework of semiotic cultural psychology and the affective-semiotic regulation model were used to guide the analyses (Branco & Valsiner, 2010; Valsiner, 2007, 2014). The analysis procedure was implemented in two phases, guided by a gradual and dialectical deepening of the data interpretation process:

### Phase 1. Preliminary Reflective-dialogical Analysis

A preliminary analysis of all the interviews was conducted to identify common themes and significant experiences of the participant and to develop initial understandings of the research problem. This process involved reflective dialogue with the therapist and was informed by the field notes.

### Phase 2. In-depth Semiotic Analysis

After transcription, the entirety of the participant’s verbalizations was analyzed using Atlas Ti software. All interviews were coded to examine and compare the data, allowing for the identification of categories. For the presentation of the results, quotations^1^ were selected after analyzing their content and contrasting them in terms of the analytical depth they could provide. The Atlas Ti software facilitated the examination of the recurrence of information and the comparison of the content of the quotations. Furthermore, a joint and reflective discussion process among the researchers helped identify the affective charge of events by connecting the interview content. The affective-semiotic regulation model offered essential analytical tools to pinpoint such affective dynamics. For instance, the participant’s choice of words and her descriptions of experiences that were diffuse or difficult to articulate in language indicated moments in which the hypoglycemic symptom acquired an intense affective charge and a central role in constructing her life experience.

### Presentation of Results

We choose to first present the general context of the case and then introduce four questions inspired by the analytical categories. These questions arise from a logical process whose purpose was to unravel the affective semiosis of the hypoglycemic symptom in the participant’s case and are based on categories derived from her deep understanding. The chosen presentation of the results chooses to be consistent with the interest of focusing on the participant’s experience. Moreover, this strategy allows us to show the semiotic transformation of the physiological experience of the symptom into a broader, hyper-generalized affective field, as will be seen below. Table 1 (Categories of analysis and presentation of results) presents the categories, their definitions, and the corresponding question from the case presentation.

**Table 1:** Categories for analysis and presentation of results

Analysis category	Definition	Question for case presentation
Discovery of hypoglycemic symptom	Initial affective-semiotic processes in the reading and understanding of the hypoglycemic symptom that are fundamental to constructing meanings about the experience from the outset.	What role does the semiosis of the hypoglycemic symptom play in the moments following the diagnosis in Lucia?
Experience of the symptom and experience of the body	Integration of the symptom into bodily experience through an affective-semiotic dynamic that transforms the perception of the body.	What do the processes of affective semiosis of the hypoglycemic symptom reveal in Lucia and how do they influence her perception of her body?
Expressions of agency	Ways in which a sense of agency is expressed, in varying and specific situations, as individuals navigate the tensions and ongoing processes of interpreting their body and context, while living with the hypoglycemic symptom.	What expressions of agency emerge in the affective semiosis of the symptom as Lucia experiences the tensions and existential dimensions of living with diabetes?
Appropriation of the disease and construction of life projection.	Semiotic dynamics through which the experience of illness is incorporated into bodily experience, influencing the perception of oneself and the projection towards the future.	How does the discovery of bodily signs lead Lucia to appropriate her illness, and what implications does this have on the construction of her life projection?

Note: This table was elaborated by the authors

### Ethical Considerations

The study complied with the ethical commitments outlined in Law 1090 of 2006, which guides the practice of psychology in Colombia. Lucía’s confidentiality, privacy, and well-being were ensured. All names and identifying details have been changed. Informed consent was obtained, ensuring the participant’s understanding of the purpose and implications of the study. She had the right to express her willingness to continue throughout the interview process. The research procedure included an individual meeting with her to provide feedback on the investigative process, fostering a respectful and supportive environment for her participation.

## Results

### General Context

Lucía is a 27-year-old woman at the time of her participation in the study. She lives with her family (father, mother, and two siblings) in a middle-class urban environment. She has no history of organic diseases, although she mentions having experienced symptoms of discomfort since the age of 10, which she later associates with an episode of Diabetic Ketoacidosis. She was diagnosed at the age of 12, with surprise, as she was unaware of the existence of T1DM. Lucia has not developed long-term complications in vital organs related to the management of the disease. She is pursuing her undergraduate studies in a School of Education.

### What Role does the Semiosis of the Hypoglycemic Symptom Play in the Moments Following the Diagnosis in Lucia?

The semiosis of the hypoglycemic symptom, as seen in Quote 1, reveals that for Lucia, being sick is primarily defined by the experience of the symptom itself rather than by the diagnosis of T1DM. This suggests that the hypoglycemic symptom plays a central role in her subjective experience of illness rather than the abstraction of the diagnosis. Before the diagnosis (See Quote 5), the bodily sensations (“I don’t feel right in the world” or “I disconnect from the world”) show a process that she did not understand and was beginning to discover, which suggests that these sensations previously had a more diffuse interpretative framework. However, after the diagnosis (See Quote 6), these episodes acquire a somewhat clearer and more immediate meaning (appreciable in the present tense as Lucia speaks), shaping her experience around the fear (“fear for life”) and the vulnerability that hypoglycemia provokes. It seems that the more concrete, bodily, and affective signs of hypoglycemia tend to structure the experience of the disease. The fact that Lucia did not experience an immediate emotional reaction to the diagnosis, but rather that this reaction emerged “years later,” reveals how the meaning of the disease is formed and evolves until certain bodily events and their affective implications begin to become present in everyday experience.

*No*,* see*,* that’s the strange thing*,* I mean*,* she got it very hard (mother) and then I got it hard*,* but I got it hard after a few years*,* I mean*,* after a few years when I*,* well*,* when I realized that for example I took care of myself*,* that I exercised*,* and that I ate healthy and that I still had low blood sugar. (Quote 1)*

In Quote 1, Lucia relates how “sugar lows” (hypoglycemia) defy the expectation that taking care of one’s health (through exercise and proper nutrition) should provide a sense of well-being. This moment of rupture is key in the semiosis of the hypoglycemic symptom because the sign system that Lucia constructed, where “eating well” and “exercising” should lead to a sense of well-being, breaks down in the face of the bodily paradox that these same actions can result in the discomfort of hypoglycemia. This contradiction causes a crisis of interpretation regarding how Lucia perceives herself in relation to her body. The sense of agency and control that self-care should grant her conflicts with the reality of the disease as her body does not respond in a predictable way, turning into an ambiguous and polysemic terrain.

Quote 2, in turn, shows how the disease is inserted into social dynamics and how Lucia begins to be perceived by her environment. Comments such as “why are you so sick” reflect the social judgment of the disease, which affects her self-image and reinforces an identity of fragility. The disease thus becomes a visible sign that not only communicates an internal imbalance, but also generates an affective response in others.

*The fact is that I remember that that same classmate once*,* once*,* on the same day of the water issue*,* she came to me and said*,* “oh*,* why are you so sick*,*” she came to me and said this*,* and I came to her and I said*,* hmm*,* I mean she said*,* “why are you so sick”*,* because I was missing a lot of school and I came to her and I said no*,* I*,* it’s not that I*,* I mean I came to her and I said*,* “what’s wrong is that I already have the disease. (Quote 2)*

By saying, “I already have the disease,” Lucia incorporates diabetes as a constitutive element of her identity. Here, diabetes becomes an identity sign that is now part of who she is. The word “already” in the sentence suggests a sense of irreversibility and resignation. It implies that diabetes has entered the realm of the permanent, affecting how she perceives herself and how others see her. The statement shows that diabetes is not only a condition Lucía lives with, but also a defining aspect of her identity, which she continuously shapes through her social interactions, including in the school context she mentions.

The richness provided by Quote 3, allows us to appreciate a continuous decision-making process, in which every aspect of Lucia’s treatment (insulin dosages, administration times and food intake) depends on constant interpretation of bodily signs and contextual circumstances. This daily management reflects the semiotic complexity of living with diabetes, where the body, insulin and food become signs that must be continuously interpreted and negotiated, undoubtedly causing her a high level of stress.

*Novorapid is the insulin that you use*,* I use it for breakfast*,* lunch and lunch but for example if I know that I am going to have very little for lunch*,* or I am going to eat very little I can’t inject it because then it goes down all at once*,* that is*,* it is an insulin that*,* let’s say I am going to have lunch*,* right? and Novorapid I inject it only*,* I am supposed to inject it in the morning and at night*,* but I only inject it at night because if I inject it in the morning and let’s say I had a very busy day*,* I sometimes plan things during the day and they turn out differently*,* so if I plan that during the day I am going to have breakfast*,* lunch*,* lunch*,* blah blah*,* and I inject the sl(…)*,* that is to say Le(…) Levemir which is the slow acting insulin*,* I inject it in the morning and at night. Levemir*,* which is the slow one*,* and let’s say a last-minute job comes up and I did not have time or what*,* I went for a walk there and I didn’t have time so I could not eat*,* or I could eat but just a little*,* and if I injected myself with the slow one then my sugar would go down all day*,* so by only injecting myself at night*,* at night is good because if it goes down at night then I get up and eat something with sugar (….) Levemir but only at night… Levemir but I only inject it at night*,* and Novorapid I inject it in the morning*,* afternoon and evening*,* depending on what I am going to eat*,* if I know that*,* for example*,* if I have pancakes for breakfast one morning*,* bread has sugar in it*,* then I must inject more. (Quote 3)*

Lucia mentions how her daily plans often change unexpectedly. Here, the *uncertainty and constant change in her daily life interfere with the predictability that diabetes treatment requires.* This means that the cues that guide her treatment (such as insulin doses and expectations about the food she will consume) become unstable and require dynamic adjustments, in which Lucia must create new ways of interpreting and responding to changes in her environment. Rapid insulin, slow insulin, food, and the body are intertwined in an interdependent set of signs that Lucia must constantly read.

Lucia’s body is an active semiotic source that reacts to food, insulin doses, and physical activities. This aspect of Quote 3 shows that the symptoms of diabetes (such as hypoglycemia or hyperglycemia) become bodily signals that must be interpreted carefully. When Lucia says that, if she does not have time to eat enough, her sugar may drop due to slow-acting insulin, she highlights the temporary and dynamic nature of bodily signs. The symptoms of the disease are not static but are constantly evolving and require her to be attentive to how her actions - or lack thereof - can destabilize that balance. Diabetes introduces a new temporal relationship between Lucia and her body. Control and regulation are based on anticipatory signs. Lucia responds to what her body feels in the moment and must also anticipate how her sugar levels will change based on insulin doses and meals. She is in the process of semiotic interpretation, where she must imagine or foresee possible outcomes from her decisions in real time. This generates a constant tension stemming from the significant responsibility she must assume to avoid the appearance of serious symptoms of discomfort, leading Lucia to experience anxiety due to the lack of control over her own body, since there are many -and sometimes undetectable- indicators of how her body will react at every moment in her life.

The daily management of diabetes, as described by Lucia, therefore, has strong affective implications. Each adjustment in her insulin dose or in her food is linked to a sense of loss of control and freedom. The reference she makes to the need to adjust to the demands of treatment “I only inject it at night and let’s say I had a very busy day…” points to how her routine, rather than being one of free choice, is mediated by the needs imposed by the disease. This aspect highlights the conflict between agency and constraint, where Lucia is forced to constantly negotiate between her desires or plans (such as having an active day or eating something she likes) and the demands of treatment.

Lucia’s case allows us to note, as seen in Quote 4, that the semiotic process of the hypoglycemic symptom involves the active interpretation of information received from different sources (specialists, internet, media), giving rise to a constantly developing knowledge about the disease.

*I am 24 years old*,* and I feel that every year I discover something new about my disease*,* that is*,* it is something that you don’t know from one day to the next*,* and you don’t have to know it from one day to the next*,* they are things that you learn little by little and the most important ones are the first years. (Quote 4)*

By saying that “every year I discover something new about my disease " she describes a continuous learning process that is not simply cumulative but is deeply articulated in the affective experience of living with diabetes. This constant discovery has a double implication: on the one hand, it reaffirms that her knowledge of the disease is under permanent construction; on the other hand, it reveals that she cannot achieve absolute control over her body and condition. This state of “continuous learning”, as she calls it, has a significant affective impact that can be associated with uncertainty, exhaustion and frustration. Living in this constant learning phase not only affects her daily life, nor does it imply merely facing the immediate challenges of the disease, but also generates questions about her future in different aspects of her life as a woman who will live with a disease that does not provide her with security or familiarity. Diabetes can become a key factor in her professional, emotional and social projection. This “continuous learning” seems loaded with ambivalence. While it gives her greater knowledge, it also reminds her of her limitations and the impossibility of having absolute control over her body and her life.

### What do the Processes of Affective Semiosis Related to the Hypoglycemic Symptom Reveal about Lucia, and How do they Influence her Perception of her Body?

In Quote 5, the hypoglycemic symptom plays a disruptive role in Lucia’s body, generating intense physiological responses that trigger an affective and cognitive alarm system. The loss of internal homeostasis triggers an affective expansion in which the immediate discomfort (dizziness, weakness) generates an emotional reaction that extends to a wider field of meanings. Hyper-generalization of affect is manifested when physical discomfort and anxiety expand and touch deep aspects related to vulnerability and control over life. The initial rupture in the body generates a series of associations that not only address the present moment, but also evoke past experiences of lack of control and future anticipations.

*When that started happening to me*,* I understood that even if I take care of myself*,* even if I go on a diet*,* my life is going to be very complicated because my blood sugar is going to go down*,* even if I am on a minimum dose of insulin and I follow my diet very well*,* my blood sugar will start to go down and down*,* and when you have low blood sugar you tend to have*,* well*,* at least I tend to feel very strange things*,* I tend to feel like I am not in the world*,* sometimes it happens to me when I have low blood sugar*,* like I am not in the world*,* it is like*,* it is like I am disconnected from the world. (Quote 5)*

The expression “I am not in the world” indicates that the physical discomfort of the hypoglycemic symptom, initially tied to the body, broadens and reaches more abstract and complex layers of experience. Lucia experiences a disconnection not only from her body but also from her surroundings and sense of identity. This suggests that the symptom blurs her ability to anchor herself to a coherent sense of self and the space she inhabits. The phenomenon also has deeper implications: as her body becomes dysregulated, her capacity to maintain a sense of stability in the world diminishes. The symptom, therefore, not only conveys physical discomfort but also a rupture in the system of signs that enables her to situate herself in everyday life. The hyper-generalization of affect is also evident in the expression “I am disconnected from the world”, which exemplifies a diffuse experience. Lucia not only feels that her body fails, but this sensation translates into an experience of existential disorientation. This disconnection is diffuse in how it is expressed, as it simultaneously affects her body, sense of self, and perception of her surroundings. It involves the destabilization of multiple levels of her experience, making it difficult to circumscribe or delimit.

Quote 6 also shows that the symptom of hypoglycemia acquires significance not only because of its physical effects but also because of its capacity to hyper-generalize affect. A interwoven set of meanings is observed that links what she feels in her body (physiological activation) with what is the object of her thinking and allows her to make immediate decisions regarding the correction of glucose levels (emotions) and the general yet diffuse feeling of vulnerability, distress, and disconnection (hyper-generalization).

*When it goes down I feel*,* I get cold*,* I start to feel like a fear*,* like a*,* I mean*,* I’m normal but I start to feel like a fear for life*,* I don’t know*,* it’s a*,* oh it’s something so strange*,* that’s why I try*,* that’s why*,* I say that when it goes down it’s more dangerous because look*,* it goes up and I*,* and you control it with water*,* insulin and now*,* it goes down*,* I mean*,* look*,* when it goes up*,* it makes you very thirsty*,* I mean you feel like a headache*,* but when it goes down I start to feel like I am afraid of the world (….) I feel like*,* it’s like*,* when it goes down*,* I start to feel like I am afraid of the world (…) I feel like*,* I feel like*,* I feel like*,* I feel like*,* I feel like I am afraid of life. I feel like*,* I arrive like*,* I mean*,* like I said*,* I can’t find myself*,* like everything scares me*,* like anything*,* I mean*,* and someone raises their voice*,* and my heart starts to race*,* oh*,* that’s horrible*,* that’s why I prefer it to go up. (Quote 6)*

It can also be observed that Lucia makes decisions based on this semiotic framework, which leads her to opt for hyperglycemia over hypoglycemia. Hypoglycemia not only triggers physiological responses, but also provokes a series of affective reactions that Lucia interprets as a general threat to her well-being and sense of identity. In contrast, Lucia prefers hyperglycemia because, although she also finds it uncomfortable, it does not trigger the same level of existential distress. Simultaneously, a sense of existential time emerges, in which the effect of hypoglycemia is not limited to an accumulation of events or an association between past and present experiences. Instead, it becomes central for Lucia, with a logic that seems to act in a more disruptive and transformative way, profoundly affecting Lucia’s decisions about her treatment in the context of her overall experience of loss of control and vulnerability.

The episodes described as the inability to recognize the environment or the motor and cognitive difficulties (“daze”, “kind of gawk”, “dizziness”, “clumsiness”) point to an alteration in Lucia’s dialogue with the body. This can be observed in quote 7, which shows the activation of signs that communicate an experience of strangeness, where the body is presented as autonomous, affecting identity and daily actions. The hypoglycemic symptom operates at multiple levels of affective organization. At the level of bodily sensations, Lucia experiences “dizziness”, “clumsiness”, and “mixed up head”, reflecting general maladjustment in her body.

*I have to take my sugar six*,* seven times a day*,* to make sure I don’t have a low*,* to make sure I don’t have a high*,* to make sure nothing bad happens to me*,* and so I have to check my sugar at the university*,* and if my sugar drops*,* I have to eat in the middle of class*,* or I have to leave class*,* these are complicated things*,* and what is complicated here is not only that I have to go out to eat to raise my blood sugar*,* it is not just that*,* ‘t’s that I end up feeling sick. It is not that your blood sugar got low*,* and you ate candies and that’s it. No*,* that will only happens*,* that will happens in the movies*,* but when a diabetic really has low blood sugar*,* he ate sugar*,* and even if his blood sugar goes up*,* even if it stabilizes*,* one is left with a certain dizziness and with a certain feeling*,* one is left with a kind of daze*,* one is left with a kind of daze*,* one is left with a kind of gawk*,* one’s head is left as if it is all mixed up*,* one is left as if*,* as if clumsy*,* one is left as if*,* what they are talking about at that moment is soon forgotten*,* and you are not concentrated on what you are doing because you are feeling bad*,* because the body is going through a sudden change. (Quote 7)*

Lucia’s description of her state even after she has stabilized suggests a disconnection between the physical sensation of having controlled her sugar and the ongoing affective discomfort. The clumsiness and lack of concentration she describes after an episode of hypoglycemia are not merely physical states but carry an affective dimension that makes her feel disconnected from her surroundings, reinforcing a perception of the body as a fragile and ever-changing. This process is also linked to the tensions that arise in the interaction between the body, the social context, and the sense of identity. Lucia mentions how these situations affect her performance at the university, where she must withdraw or eat during classes. In this sense, the symptom alters not only her body but also her social role and identity as a student. The impact on her physical state also modifies her perception of agency since she cannot fully control or predict how her body will react, leading to constant vigilance and effort to prevent the consequences of the symptom. Here, it’s crucial to note that the response to treatment has a symbolic component that seeks not only to correct glucose levels but also to restore an affective balance that has been disrupted.

### What Expressions of Agency Emerge in the Affective Semiosis of the Symptom as Lucia Experiences the Tensions and Existential Dimensions of Living with Diabetes?

Quote 8 reveals how the processes of affective semiosis related to the hypoglycemic symptom are presented as a space of constant interpretation within the body, mediated by affective signs that vary in intensity. Lucia gives meaning to these experiences by comparing different bodily states (such as sugar spikes and lows), informed by both her own sensations and the medical information provided by her endocrinologist.

*That’s the good thing*,* you can raise your sugar without eating candies*,* but when I’m at the University I have to raise my sugar super-fast because the faster I raise it*,* the faster the side effects will pass*,* and the day before yesterday I also had lunch*,* it was like three hours*,* that is*,* not even two hours passed*,* in fact*,* an endocrinologist would tell you*,* sugar at that level can make you blind*,* because the endocrinologist has emphasized to me that she prefers me to have a sugar spike*,* that is*,* she prefers me to have sugar spikes rather than low blood sugar. (Quote 8)*

In Quote 8, Lucia is in a state of constant vigilance over her body. The urgency to stabilize sugar levels generates a series of immediate emotional and cognitive responses. The fact that she must “get her sugar up super-fast” in college not only reflects a pragmatic response to the symptom, but shows an understanding of the interplay between body, social context, and identity. It suggests that the symptom not only alters the body, but also the sense of agency and her social role as a college student. Furthermore, the semiosis of the symptom suggests a relationship with the body that is continually modulated by the fear of long-term effects (such as blindness). Her perception of her body is also revealed as a space of permanent negotiation between what she feels and what medical experts advise. In her relationship with her body, she continuously adapts to the immediate demands imposed by diabetes and her occupations (such as the university). The body acts as a semiotic vehicle that mediates between the organization of her daily life and experiences that involve constant rediscovery and adaptation. At the same time, Lucia seems to internalize the voice of her endocrinologist, but not passively. Rather, she reinterprets it, aligning it with her own desires and needs regarding her condition. One could argue that Lucia uses medical discourse as a semiotic tool to validate her preference for avoiding hypoglycemic symptoms. By stating that her endocrinologist “prefers me to have sugar spikes rather than low blood sugar”, Lucia is justifying her daily management of diabetes, aligning this external voice with her own emotional processes and understanding of the disease. This may function as a self-care strategy that allows her to express her agency and manage the condition according to her priorities. Through this strategy, she creates a space where she feels more secure and in control of her illness’s symptomatic expressions and the uncertainty they bring. Lucia not only avoids physical and cognitive debilitation but also positions herself with a sense of agency, viewing medical recommendations as adaptable to her reality.

The analysis of Quote 9 allows us to observe a deep and dynamic connection between affectivity, the body and the self-regulation that Lucia deploys to manage the hypoglycemic symptom. Her statement that “the body panics” reflects an immediate emotional state in response to hypoglycemia, an affect that functions as an alarm signal. This emotional response not only leads her to “eat everything”, but also prompts an internal dialogue as an attempt at self-regulation. It is interesting how this internal dialogue connects with her bodily perception, almost as if it were a negotiation between what she feels and what she knows she must do.

*When you have low blood sugar*,* the body panics*,* that is*,* the body panics so I start eating everything. You see*,* it has happened to me that sometimes when my sugar gets low*,* I start talking to myself*,* that is*,* I always feel like I have to be the one who controls myself*,* the one who helps me*,* so I start talking to myself and I start telling myself well*,* let’s calmly look at what we are going to eat and obviously you are not going to feel good right away*,* but you have to wait minutes and minutes with that discomfort until you finally feel good*,* because that is the problem*,* of course*,* I start eating and eating*,* and I realize that I still feel very bad*,* I keep on eating*,* I mean I think “no*,* I still feel bad*,* that is*,* my sugar will continue to go down”. Or when it happens at dawn*,* I feel like*,* “no*,* I want to keep on sleeping*,* I am very sleepy*,* so let’s eat a lot so I can go to bed*,* and I don’t have to get up anymore”. (Quote 9)*

Lucia’s case seems to reveal a process of semiosis in which the body, confronted with the symptom of hypoglycemia, becomes an object of constant interpretation. She deciphers it through her bodily sensations and the relationship she establishes with the symptom, as if it were a form of body language that she interprets minute by minute. Of course, this constant reading of the body is neither neutral nor free of affective costs. While she interprets and responds to the signs her body emits, Lucia appears to experience a sense of emotional and physical exhaustion. The phrase “you have to wait minutes and minutes with that discomfort until you finally feel good” suggests not only the immediate physical discomfort, but also the exhaustion of being trapped in a cycle where relief is never instantaneous or complete. This discomfort extends beyond the momentary symptom, affecting her ability to concentrate, rest, and, ultimately, to live without being constantly vigilant of her body.

The act of “eating a lot” to avoid future nocturnal interruptions reveals a deep desire to free himself, even temporarily, from the constant control that the disease demands. This decision, which stems from the desire to “don’t have to get up anymore”, reveals a longing for liberation from the cycle of vigilance and the consequences of the symptom. It seems that Lucia wants to regain autonomy over her life, but this desire clashes with the inescapable reality of her body, which continues to impose its own logic reminding her of her vulnerability. At the same time, Lucia not only responds to her body but also engages in a dialogue with it, interpreting its signals and using her own agency to regain some control over her discomfort.

Quote 10, in turn, reveals that Lucia has had experiences with her illness that have shaped her decisions on how to manage it through medical therapeutics. This decision-making involves strong emotions in an active discovery process of the impossibilities of healing. Her interpersonal relationships -with her father, for example- have involved tensions and intense affective processes.

*The truth is that I am not going to make a remedy if I do not have faith at the time of doing it*,* if one is going to make a remedy and one already has the thought that it is not going to cure the diabetes*,* then what’s the point? But yes*,* according to him (the father)*,* that is the funniest thing*,* that he does not say that it is a remedy to improve my lifestyle but he tells me that it is a remedy to cure diabetes so that is what it is for me*,* to me when he tells me that it is a cure*,* it makes me angry*,* I mean it makes me angry that he is playing with something like that*,* because for a long time I cried until I resigned myself to the fact that I had no cure*,* maybe later there will be a cure*,* maybe later*,* I do not know*,* they will create an artificial pancreas*,* I do not know*,* but I know that with food*,* that is*,* with home remedies it will not be cured. (Quote 10)*

By expressing that making a remedy is pointless if she doesn’t believe in its effectiveness, Lucia demonstrates that her therapeutic decisions are based not only on what others propose to her but on a personal and affective evaluation. The idea of “having faith” in a remedy goes beyond medical logic, revealing the intersection between knowledge, belief, and affect. Lucia’s decision not to follow the remedy her father suggests is mediated by the certainty she has developed that diabetes is not cured by home treatments. This decision-making process is deeply intertwined with emotions such as anger and resignation.

Lucia’s relationship with her father is a context where affective tensions clearly emerge. The fact that he insists on talking about a “cure” for diabetes through home remedies reflects a profound disengagement from Lucia’s experience with the disease. It revives a hope that no longer makes sense to her. Now, the rage that Lucia feels is not just a superficial reaction; it reflects a long and complex emotional process in which she has had to accept the impossibility of a cure and resign herself to living with the disease. By rejecting the home remedies her father suggests, she is not only making a science-based decision but also emotionally protecting herself from expectations that will not be fulfilled. This decision-making process also places her in a position where she must deal with the incomprehension of those around her.

Finally, in Quote 11, Lucia expresses an agency that arises in a space of uncertainty. The fact that she “never knows” whether carbohydrate counting will make a “positive difference” in her life reveals a sense of agency mediated by contingency. Lucia’s agency is not an absolute agency, but an ability to make decisions and execute actions even when the outcomes are uncertain.

*Yes*,* I also have to try*,* because one never knows that the carbohydrate count can really make a positive difference in my life*,* because look*,* even to this day*,* I’ve had the disease for what*,* nine*,* ten*,* thirteen years now*,* and it still happens to me*,* it happens to me a lot*,* I had lunch*,* I injected my insulin*,* I tried to calculate super well and still two hours later my sugar level falls to the floor. (Quote 11)*

In Lucia’s case, there is a clear manifestation of the tension between the desire for control and the reality of the impossibility of absolute control. She injects insulin, accurately counts carbohydrates, and follows a set of medically informed self-care practices. However, saying, “…two hours later my sugar level falls to the floor”, is a statement that exposes the gap between the intention to control and the adverse outcome. This gap highlights the awareness that her agency, although present and active, is not sufficient to eliminate the unpredictability of her body (See also Quote 4). Now, Lucia’s sense of agency also resides in her reflexive ability to confront the tensions between her efforts to manage the disease and the changing nature of her condition. By mentioning that she has had the disease “thirteen years now”, Lucia underscores the cumulative dimension of her experience, suggesting that her agency is not defined by a single event or moment but by the continuity of her attempts, mistakes, and learning over time. However, her vulnerability to symptom also becomes a space where her agency is redefined and reaffirmed. Persistence reflects an agency understood as the capacity to deal with uncertainty and to continue acting despite unforeseen outcomes. The phrase “*look*,* even to this day*” indicates critical reflexivity in Lucia, a moment in which she evaluates her agency in retrospect. Through this evaluation, an affective semiosis of treatment emerges as an ongoing engagement. In this quote, Lucia also reveals a key existential dimension of her experience with diabetes: the acceptance that her illness is an inseparable part of her daily life. Thirteen years with diabetes means not only a trajectory of learning how to manage the disease, but also a profound affectation of her sense of self, as the constant need for adjustment in treatment reflects the fact that the disease is not something external that she can completely control, but something that continuously shapes her daily life.

### How does the Discovery of Bodily Signs Lead Lucia to Appropriate her Illness and What Implications does it have on the Construction of her Vital Projection?

Through the semiosis of the bodily signs, Lucia continually appropriates her illness. In Quote 12, Lucia compares her experience to that of a person without diabetes, highlighting how her body behaves differently and how that difference forces her to live with constant vigilance. This comparison with a person without diabetes not only highlights the uniqueness of her situation, but also evidences her appropriation of the disease: the recognition that she cannot “allow” herself certain things that others can. This continuous discovery of bodily signs leads her to appropriate her body in a very concrete way: she needs to read it and act on the signals she constantly receives, which also shapes her ability to project herself into the future.

*Do you see the difference? that a person who does not have a disease*,* I take the example like this*,* in a lost jungle*,* if a normal person gets lost in a jungle*,* well*,* they wait for the rescuers but if you cut yourself*,* well*,* if you did not eat*,* well*,* but if I do not eat*,* I cannot go without eating*,* I cannot allow myself to say I am not going to eat tonight*,* no! my blood sugar goes down*,* and also if you do not eat*,* your blood sugar goes up (emphasis added)*,* in other words*,* you have to have a balanced diet. (Quote 12)*

The constant need for a glycemic balance not only regulates her physical health, but also regulates her time, energy, and life plan. Now, the process suggests a sense of differentiation on Lucia’s part. Her body, in its difference, imposes limits that are not only physical but also existential. The affective semiosis here manifests in how Lucia seems to live these differences as part of her identity. It is not just a matter of learning to cope with illness but of living with the feeling that there is always something uncontrollable, which prevent agency not to be fully exercised.

In Quote 13, in turn, it can be observed that Lucia expresses how the experience of the disease allows her to relativize concerns such as economic ones. The discovery of the bodily signs of diabetes, with its physical and emotional discomfort, has led her to value life in a different way, giving her the ability to place her illness at the center of her life experience.

*Well*,* the truth is that I don’t usually think much about that*,* I only know that*,* yes*,* of course*,* when I become a teacher I will earn a little*,* but the truth is that I feel very calm because I think that*,* I don’t know*,* I think that is one of the reasons why*,* one of the reasons that make you value life so much is illness*,* so since I know and I have experienced what it is like to be sick or very sick then that doesn’t make me feel frustrated*,* like “oh*,* I will earn very little!” (Quote 13)*.

Lucia now situates her vital projection in a broader relationship with her body and her illness. Illness becomes here a point of reference to relativize other problems. Still, the discovery she has made about her physical vulnerability (“I have experienced what it is like to be sick”) carries an emotional weight that is not simply reduced to a more philosophical appreciation of life. Lucia is attempting -in a way that is not closed- to integrate her experience of illness into a broader view of life. Still, this integration is marked by an ongoing negotiation between recognizing her limits and the desire to find meaning in them. Although she claims to feel “calm” in valuing life because of her experience with illness, the expression of this calmness may be a form of mitigating the discomfort (see Quotes 4 and 11) or affective containment. “Being calm” may be a process of conscious meaning-making that seeks to generate a state of well-being under construction, where Lucia continues to search for meaning in her illness.

Finally, in quote14, Lucía reflects on a process of existential discovery through the experience of being hospitalized. Her illness places concrete limits on her ability to study and work. The fact that she has overcome difficult moments related to diabetes becomes a key semiotic referent to face the future.

*I remember that at the time I was hospitalized I was working and studying obviously*,* I realized that if you are not healthy*,* you can’t study or work*,* you can’t do anything*,* so that’s when I thought*,* “well*,* first my health*,* and if I don’t have health I can’t aspire to other things”*,* So I feel that the disease has helped me a lot to value life*,* sometimes I have a problem*,* and I feel that I set it aside*,* or that I am not frustrated by the simple fact of knowing that the disease is complicated and that I have felt so bad with diabetes*,* and that if I have been able to overcome that*,* I have been able to go ahead with it*,* then for the rest*,* the rest I can fight for it. (Quote 14)*

In this sense, the disease shapes her perception of priorities in her life. Lucia’s case does not place meanings of victory over the disease, rather it shows processes that are partially redefined time after time to deal with the limits that her body imposes on her. The phrase “if I have been able to overcome that” seems to reflect an effort to convince herself that the disease can be managed. Still, at the same time it carries an implicit affective charge that suggests the opposite: that living with diabetes is an ongoing challenge that is not solved by a single overcoming. Lucia seems not to project herself in terms of external goals such as a job or academic success, but rather in her ability to maintain her health.

## Discussion and Conclusions

The preceding research addressed the affective semiosis processes involved in the hypoglycemic symptom in T1DM to propose its subjective implications based on the richness of information provided by Lucía’s case. We consider that this analysis brings a novel understanding to the framework of semiotic-cultural psychology -still developing and called to study affective processes- by showing how the symptoms of a chronic disease, such as hypoglycemia, function as signs capable of transforming the individual’s perception and subjective organization around their own bodies, the environment, and the interpersonal relationships that surround them. From an empirical point of view, these results are valuable because they allow us to propose how the model of affective semiosis serves as a useful theoretical framework for studying chronic conditions and their psychological and cultural implications.

What stands out most clearly is that the physical signs associated with hypoglycemia not only generate an immediate physiological response but also trigger a rapid and simultaneous reorganization of all levels of affective semiosis. The initial sensation of physical discomfort activates emotions that are quickly linked to generalized meanings about the disease, which then expand to encompass broader aspects of life and identity, primarily relating to feelings of disconnection and vulnerability. These dynamic and non-linear processes illustrate how, in situations of bodily crisis, the various levels of organization of affect (sensations, emotions, generalized categories, and hyper-generalized meanings) interact, resulting in a profound transformation of experience. Additionally, these processes are characterized by the interplay between chronological and existential time, shaping the experience and understanding of the disease, which in turn impacts daily life and future projections. In situations of hypoglycemia, physical discomfort serves as merely the starting point of a more comprehensive semiotic network. Immediate bodily sensations, such as dizziness or weakness, are quickly intertwined with emotions like anxiety or fear, which in turn refer to broader meanings associated with control and vulnerability (Branco & Valsiner, 2010; Lehmann & Murakami, 2024; Valsiner, 2014).

The dynamism and complexity of the disease generate an “affective semiosis of the symptom.” This case reveals the processes by which the body, confronted with hypoglycemic symptoms, becomes an object of constant interpretation, primarily based on bodily sensations as if it were a body language that is continually being deciphered. In this way, it may bring significant loneliness to those who experience it. However, this process is also linked to the tensions that arise in the interaction between the discomfort of the body and the social role (in the case of Lucia, mainly in her role as student, patient, and daughter).

This case highlights how life projections for an individual with T1DM are often mediated by the fear of the hypoglycemic symptom. The constant need for glycemic balance not only regulates physical health but also influences time management and life planning. Hypoglycemic symptoms can raise questions about managing the uncertainty of a body that defies expectations, where adherence to medical guidance does not always guarantee a sense of well-being—defined as the absence of bodily sensations and the latent meanings they carry. Consequently, decisions regarding treatment involve not only the correction of glucose levels but also the acceptance of some hyperglycemic episodes that may contribute to a greater sense of mastery and reduced vulnerability. The disease, in turn, shapes perceptions of life priorities, prompting individuals to focus less on external goals (such as work or academic success) and more on the capacity to maintain health.

Now, the hyper-generalization of affect not only extends anxiety and fear to deeper aspects of life with the disease, but also requires affective regulation to restore some sense of control during the discomfort. However, this regulation is not a simple process since hypoglycemic symptoms are not delimited to the immediate physiological moment, as has been clearly seen. The case allows us to observe that the agency emerges in a space of uncertainty. Agency makes it possible to devise specific strategies, to reflect on the relationship between the disease and the personal future and implies the ability to make decisions and execute actions even when the results may be uncertain.

In summary, the present study made it possible to identify and analyze an important dimension of the experiences lived by people who must live with chronic diseases that effectively impact their quality of life and, consequently, their development trajectory (Zittoun, 2006, 2013). Therefore, all factors associated with experiences that tend to destabilize these people, causing them discomfort or even putting their lives at risk, should be identified and discussed with each person to assess how each one is affected through affective-semiotic meaning fields directly linked to the perception of oneself as a subject and expectations regarding the life trajectory. Only after a thoroughly investigating how each person interacts with the symptoms, the diagnosis, and the different ways in which social others relate to them, it will be possible to co-construct, with that person, effective treatment guidelines that even favor adherence processes (Cleves-Valencia et al., 2024). In other words, by working on the affective-semiotic dimension of their experiences, it will be possible to open pathways to reduce uncertainty and promote greater personal empowerment in terms of active and constructive behavior in one’s own life, minimizing the levels of anxiety and depression that more easily affect patients with chronic severe diseases.

This study represents a valuable step in understanding the affective semiosis of hypoglycemic symptoms in individuals with T1DM, offering insights that may be transferable to other chronic conditions. Future investigations should consider longitudinal designs that capture data at different stages of the disease trajectory, particularly during diagnosis and across the short, medium, and long term. Such studies could shed light on how meanings are constituted and configured over time, shaped by individual and cultural dynamics. Additionally, this study briefly explores the role of significant others in shaping the participant’s affective experiences. Further examination of these relational dynamics could provide deeper insights into how interpersonal relationships influence the lived experience of T1DM, contributing to a richer theoretical and practical understanding.

## Data Availability

Data can be asked directly from the authors.

## Footnotes

1 All quotations were translated by the authors, as these were originally written in Spanish.
